# Fake news detection based on a hybrid BERT and LightGBM models

**DOI:** 10.1007/s40747-023-01098-0

**Published:** 2023-05-24

**Authors:** Ehab Essa, Karima Omar, Ali Alqahtani

**Affiliations:** 1 Department of Computer Science, Faculty of Computer and Information Sciences, Mansoura University, Mansoura, 35516 Egypt; 2 Department of Computer Science, King Khalid University, 61421 Abha, Saudi Arabia

**Keywords:** FakeNews, BERT, LightGBM, Transformers

## Abstract

With the rapid growth of social networks and technology, knowing what news to believe and what not to believe become a challenge in this digital era. Fake news is defined as provably erroneous information transmitted intending to defraud. This kind of misinformation poses a serious threat to social cohesion and well-being, since it fosters political polarisation and can destabilize trust in the government or the service provided. As a result, fake news detection has emerged as an important field of study, with the goal of identifying whether a certain piece of content is real or fake. In this paper, we propose a novel hybrid fake news detection system that combines a BERT-based (bidirectional encoder representations from transformers) with a light gradient boosting machine (LightGBM) model. We compare the performance of the proposed method to four different classification approaches using different word embedding techniques on three real-world fake news datasets to validate the performance of the proposed method compared to other methods. The proposed method is evaluated to detect fake news based on the headline-only or full text of the news content. The results show the superiority of the proposed method for fake news detection compared to many state-of-the-art methods.

## Introduction

Social media systems have attained extraordinary levels of achievement, opened unforeseen opportunities, and been changing the way news is disseminated, produced, and consumed, thereby becoming indispensable platforms used for a variety of applications. The dramatic development of social media characteristics in various platforms made the migration to social media platforms greatly desired even by reputable/well-known news organizations and agencies.

The usage of social media is accompanied by a dramatic increase in the threats of fake news and online misinformation. Fake news is fabricated stories that have similar characteristics to news media content but differs in organizational process or purpose in an effort to deceive readers [1]. Fake news is constantly growing through social media, online blogs, magazines, forums, and newspapers, making it difficult to find trustworthy news sources. Social media has evolved into an ideal platform for anybody to manufacture, distort, and propagate fake news. Because of the ease with which information can be created and distributed. For instance, according to Facebook [2], malicious entities contributed to less than one-tenth of 1% of civic content published on the network.

In recent years, fake news has been blamed for deepening political division and party strife, and also, it has a significant effect on topics, such as vaccination, nutrition, and stock values. According to a study by Ohio State University academics [3], false news most likely contributed to the decline in Hillary Clinton’s popularity on election day. The study implies that roughly 4% of Barack Obama’s 2012 supporters were discouraged from voting for Clinton in 2016 by their belief in false news articles. Another example [4], fake rumors about Tesla buying a lithium mining caused its shares to increase by nearly $250\%$. According to [5], tweets about the COVID-19 pandemic contained inaccurate or unverified information, with $24.8\%$ and $17.4\%$, respectively. The falsity of news has a considerable (positive/negative) influence on readers. The development of effective analytical tools for online content is crucial to prevent having a negative impact on social, economic, and political life.

Manual fact-checking needs regular and manual updates by crowdsourced people or a small set of experts and also is unable to perform automated learning [6]. Machine-learning and deep-learning approaches have demonstrated accurate predictions and insights to handle a variety of complicated problems [7–9]. Developing automatic, trustworthy, and accurate solutions for detecting fake news is a hot research area. The detection of fake news is a challenging natural language processing (NLP) problem that is concerned with text classification to distinguish between fake and real. NLP has advanced significantly over the past few years. Transformer-based pre-trained language models are now the state-of-the-art approach for many NLP problems [10–12]. However, studying fake news detection using transformer-based models is still limited.

In this paper, we propose a hybrid model that combines a transformer-based architecture with a light gradient boosting machine (LightGBM) for fake news detection. Bidirectional encoder representations from transformers (BERT) are used to process the news articles and extract the text representation. BERT is one of the most effective language representation models, producing excellent results across a wide range of NLP applications. We add a LightGBM at the end of the BERT model to produce a hybrid classification that predicts “true” or “false” about news content. LightGBM is a high-efficiency gradient boosting framework that uses tree-based learning techniques. It supports parallel, distributed, and GPU learning and provides faster training speed. The proposed method is evaluated on three fake news datasets. We compare the performance of the proposed method with multinomial Naive Bayes (MNB), linear regression (LR), linear support vector machines (LSVM), and long short-term memory (LSTM) using different word embedding techniques. The proposed method achieves superior performance compared to the state-of-the-art. The main contributions of this work can be summarized as:

- We propose an automated fake detection method for both the title and the full text of news articles based on a hybrid of BERT and LightGBM models.
- The BERT model is proposed to extract a deep representation of the input texts.
- The LightGBM model is proposed to classify the BERT-based word embedding as real or fake content.
- The proposed method is evaluated on three fake news datasets and compared to traditional machine-learning and deep-learning approaches.

The rest of the paper is organized as follows. In the section “Related work”, the related works on fake news detection are discussed. The section “Proposed method” presents the details of the proposed method. The experimental results, as well as a comparison to other methods, are discussed in the section “Experimental results and discussion”. Finally, the conclusion is drawn in the section “Conclusion”.

## Related work

Various machine-learning-based techniques have been developed to detect fake news. These methods can be categorized into traditional approaches and deep-learning approaches.

The traditional machine-learning approaches, such as MNB, LR, LSVM, Decision Tree (DT), and extreme gradient boosting (XGBoost). In Ref. [13], Ahmed et al. used *n*-gram analysis with term frequency-inverted document frequency (TF-IDF) to extract features for detecting fake news. They studied and compared six different machine-learning techniques. The LSVM model achieved the highest accuracy score of 92% on ISOT dataset. However, it is unclear whether this approach can generalize well to other datasets. Similarly, in Ref. [14], the author studied five different machine-learning models with the same embedding technique. LSVM and XGBoost achieved the best results. In Ref. [15], Ozbay and Alatas applied the term frequency (TF) weighting method and document-term matrix to extract features from texts and then investigated 23 supervised models to identify fake news. DT is given the best results according to this study. Similarly, in Ref. [16], the same authors adapt salp swarm optimization (SSO) and grey wolf optimizer (GWO) algorithms instead of machine learning methods for the fake news detection problem. In Ref. [17], Kansal studied underlying writing style based on part-of-speech (POS) tags to detect fake news. The POS features are fed into the XGBoost to create the first model. An average of TF-IDF weights and Word2Vec word embeddings are fed into the multi-layer perception and then ensemble with the first model to get the final prediction. However, these methods require large amounts of labeled data and sophisticated models to achieve high accuracy. Moreover, these methods can struggle to adapt to new types of fake news, which are constantly evolving.

Deep learning is regarded as one of the hottest topics in the fields of machine learning, and artificial intelligence due to its learning capabilities from given data. Methods, such as convolutional neural networks (CNN) and LSTM, are becoming increasingly popular. In Ref. [18], Nasir et al. proposed a hybrid model of two deep-learning models: CNN and LSTM. CNN is used to extract features and then feed these features as input to LSTM to learn the long-term dependencies. Words in the text are represented as vectors using the global vectors for word representation (GloVe) pre-trained word embeddings. The method achieved better performance compared to seven traditional machine-learning methods. In Ref. [19], they introduced a deep-learning model called (FNDNet), such that the GloVe is used as a pre-trained word embedding. Three convolutional-pooling layers are used to extract features from the word embedding vectors, and then, these features are concatenated and fed into other convolutional-pooling layers followed by two dense layers for classification. In Ref. [20], Sastrawan et al. applied back-translation as a data augmentation to reduce imbalance classes and then carried out pre-processing step on the augmented data. They compared the performance of using pre-trained word embedding (Word2Vec, GloVe, and fastText) on pre-processed data. They tested three different deep-learning models, namely CNN, bidirectional LSTM, and ResNet to extract features from word embedding vectors and then detect fake news. In Ref. [21], an optimized deep-learning model called OPCNN-FAKE is proposed based on CNN. It consists of an embedding layer to create embedding vectors, a dropout layer to enhance regularization, a convolution-pooling layer to extract features and reduce the feature map, a flattened layer to produce a one-dimensional vector, and an output layer that takes the output of the previous layer and decides if the input text is fake or real. They compared their performance with Recurrent Neural Networks (RNN), LSTM, and six regular machine-learning techniques. In Ref. [22], Yang et al. introduced a CNN-based model (TI-CNN) that combines the explicit and latent features from both text and image information for detecting fake news. They collected a dataset for this purpose and compare their method to other models, such as LSTM, CNN, and GRU. Similarly, in Ref. [23], authors proposed a multi-modal coupled CNN model that fuses both text and image data to classify online news. However, these methods may not able to capture long-range contextual information. Moreover, the word embedding is not representing the context-specific information in the text.

Recently, the transformer model has emerged as one of the key highlights of deep-learning advances in NLP. In Ref. [24], authors compared five transformer models (XLNet, BERT, RoBERTa, DistilBERT, and ALBERT) to detect fake news. They run the experiments with various combinations of hyperparameters. The results showed comparable results of the transformer models. In Ref. [25], Kaliyar et al. proposed a deep-learning model (FakeBERT) based on BERT. The model is using BERT to get the word embeddings and feed it into three parallel layers of convolution with different kernel sizes. This BERT-based model showed better performance than other machine-learning-based methods. In Ref. [26], authors introduced a transformer-based deep learning model based on BART and RoBERTa to distinguish between different types of news articles. Each output of BART and RoBERTa embeddings is fed into a branch of LSTM and CNN-based architecture and then concatenated and passed through another LSTM and CNN-based architecture to get the results. In Ref. [27], Qazi et al. used an attention-based transformer model to detect fake and real news. They compared their performance with a hybrid CNN model that integrates text and meta-data. The transformer model showed better accuracy compared to the hybrid CNN model. However, many of these methods used deep-learning-based techniques combined with transformer models, which can be computationally expensive and require large amounts of training data. In contrast, the proposed method utilizes the LightGBM, which is much faster and requires less computational power.

**Fig. 1:**
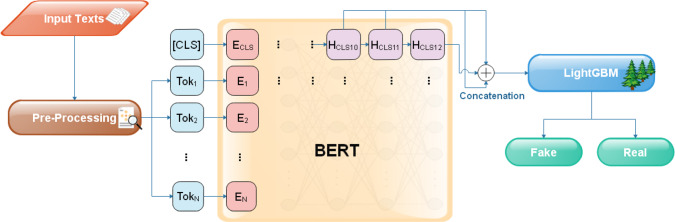
An overview of the proposed method

## Proposed method

Fake news is a growing problem in today’s society, with the potential to mislead and harm individuals, organizations, and even entire nations. Existing approaches to fake news detection often rely on manual fact-checking or rule-based systems, which can be time-consuming and limited in their coverage. In this paper, we propose a novel method based on fine-tuned BERT and LightGBM to improve the accuracy and efficiency of fake news detection. Our method leverages the power of BERT to capture complex linguistic patterns and the efficiency of LightGBM to optimize feature space and classification. By combining these two techniques, we aim to achieve better performance compared to other methods.

Figure 1 shows an overall view of the proposed system. We apply some pre-processing steps on the input text to get rid of unnecessary parts of the data. The input text is then tokenized to individual characters, subwords, and words that are good enough to represent the input data to be fed to “fine-tuned” BERT. We extract the text embedding from the special token [CLS] of the last three hidden layers of the BERT. We train the LightGBM model on the concatenated embedding vectors to get the final prediction.

### Pre-processing

In this paper, we apply several pre-processing steps to clean the input data and reduce noise. All non-alphabet characters, tags, and URLs are filtered out from the text, since they may not provide much importance to understanding the text. Numbers are deleted as they represent quantified arguments in the news context and do not generally alter the meaning of the text. Stop words (e.g., “the”, “a”, and “is”, etc.) and punctuation (e.g., “!”, “?”,“-”, etc.) are removed, because they are more frequent and provide less helpful information. Case folding is applied to reduce all letters to lowercase. Finally, we exclude the news record from any analysis, if the number of words of its full text is less than ten words.

### BERT

Incorporating a pre-trained language model attracted much attention to many NLP tasks such as paraphrasing [28], natural language inference [29, 30], named entity recognition [31] and question answering [32, 33]. BERT [10] is a pre-trained bidirectional language model based on a transformer that produces language representations by combining both left and right contexts. It analyzes input text bidirectionally from left to right and right to left. BERT is a contextual model that considers the word position in a sentence to computer the representation of each word, unlike other word embedding techniques such as Word2Vec and GloVe which is a context-free model that produces the same word representation regarding less the word position in a sentence.

BERT is using the “masked language model” (MLM) pre-training objective to achieve bidirectional representations. The MLM is randomly masking $15\%$ of the input tokens and the task is to retrieve the original token of the masked word. Moreover, the next sentence prediction task is also utilized to train the BERT model to capture the relationship between sentences by predicting the sentence that comes after the current sentence. Two large corpora of unlabeled text: BooksCorpus (800 million words) and English Wikipedia corpus (about 2.5 billion words) are used for the pre-training of the BERT model. Before processing the input to BERT, the input sentences are tokenized to individual characters, subwords, and words that are good enough to represent the input data. BERT is using a fixed size of 30,000 token vocabularies. Special tokens [CLS] and [SEP] are added at the beginning and end of each sentence, respectively. WordPiece embeddings is a tokenization algorithm used by BERT. It works by first checking the whole word if it is in the vocabulary list, it returns the corresponding word embedding vector. If not, it breaks up the word into the best-fit subwords and eventually individual characters that are in the vocabulary list. So that the original word representation will be as an average of all subword embedding vectors. The output representation for a given token is computed by summing the position and segment embeddings to the token embedding.

BERT is a transformer-based model [34] which is different from RNN. A transformer is a deep-learning model that uses the attention mechanism to process all the input sequences at once without the need for RNNs. The attention mechanism examines the relationship between words, regardless of the places of words in a sentence. RNNs process input in a sequential manner, word by word, so it is difficult to parallelize the computation. This makes the training of RNNs inefficient when dealing with long sequences.

The original transformer model [34] is used in machine translation. It has two parts an encoder and a decoder. The encoder is producing an embedding representation for each word depending on their relationship to the other words in the sentence. The decoder takes the output embeddings of the encoder and turns them back into output text. BERT makes advantage of the transformer’s encoder only, since its purpose is to develop a model that can work well on a variety of NLP tasks. Using the encoder part, the BERT is able to encode semantic and syntactic information in the embedding, which is required for a variety of jobs.

The base architecture of BERT adapted in this paper consists of a number of encoder layers ($L=12$) and each layer has a hidden size (*H*) of 756 units, and the number of self-attention heads $(h=12)$. The total number of parameters is 110*M*. The input embeddings are passed through multiple layers of self-attention and feedforward networks called TransformerBlock. The output of each layer is denoted by $H_{l}$, where *l* is the layer index

1 $$\begin{aligned} H_{l} ={\text {TransformerBlock}}(H_{l-1}). \end{aligned}$$

The fundamental operation of the *TransformerBlock* is to compute multi-head attention as follows:

2 $$\begin{aligned} {\text {MultiHead}}(Q,K,V) = {\text {Concat}}({\text {head}}_1,\ldots , {\text {head}}_h)W^O,\nonumber \\ \end{aligned}$$

where

3 $$\begin{aligned} {\text {head}}_i = {\text {Attention}}({{QW}}_i^Q, {{KW}}_i^K, {{VW}}_i^V), \end{aligned}$$

where *Q*, *K*, and *V* are the query, key, and value matrices, respectively. $W_i^Q$, $W_i^K$, and $W_i^V$ are learnable weight matrices for the *i*-th head. $W^O$ is a learnable weight matrix to map the concatenated attention outputs back to the model dimension. The output of multi-head attention is then fed through a feedforward network layer, which applies a non-linear transformation to the attention output. The residual connection and layer normalization are used to stabilize the training process and improve the overall performance of the network.

### BERT fine-tuning

One of the important characterizations of BERT is transfer learning, which allows using a previously trained BERT on large datasets in another specific task using the fine-turning technique. Here, the fine-tuning is adapting the BERT parameters on the fake news detection task.

BERT represents the whole sequence using the last hidden state $H_{{\text {CLS}}_{L}}$ of the first token $[{\text {CLS}}]$. To fine-turning BERT, a fully connected layer with a softmax classifier is added to classify vector $H_{{\text {CLS}}_{L}}$ into real or fake. The dimension of the fully connected layer is [768, 2]. The output layer of the model predicts the probability of label *C* using the softmax function

4 $$\begin{aligned} p(C\mid H_{{\text {CLS}}_{L}})={\text {softmax}}(WH_{{\text {CLS}}_{L}}), \end{aligned}$$

where *W* is the parameter matrix of the newly added fully connected layer. All the parameters of BERT as well as *W* are fine-tuned by minimizing the negative log-probability of the correct label.

### Sentence representations

After fine-tuning, we extract fixed features’ representation of the input sentences from the BERT to create a fake news detection model using the LightGBM. There are many ways to get feature embedding from the BERT. Most of them focus on utilizing the embeddings of ${\text {CLS}}$ token in the last set of the encoder layers. In this paper, we concatenate $H_{{\text {CLS}}}$ embedding vectors of ${\text {CLS}}$ for the last three layers into one feature vector. The feature representation, *E*, is calculated using the following formula:

5 $$\begin{aligned} E={\text {Concat}}(H_{{\text {CLS}}_{L}},H_{{\text {CLS}}_{L-1}},H_{{\text {CLS}}_{L-2}}). \end{aligned}$$

### LightGBM

Gradient-boosting decision tree (GBDT) [35] is an ensemble model of weak learners based on decision trees that are trained sequentially. GBDT learns the decision trees in each iteration by fitting the negative gradients (also known as residual errors). The GBDT model *f*(*x*) can be expressed as a sum of decision trees

6 $$\begin{aligned} f(x) = \sum _{m=1}^M \gamma _m D(x;\theta _m), \end{aligned}$$

where *M* is the number of trees in the model, $\gamma _m$ is the learning rate, $D(x;\theta _m)$ is the m-th decision tree, and $\theta _m$ are the parameters of the tree. The m-th tree is trained to predict the residual error by minimizing the loss function ${\mathcal {L}}$ with respect to the tree parameters $\theta _m$

7 $$\begin{aligned} \theta _m = {\text {*}}{arg min}_\theta \sum _{i=1}^{N} {\mathcal {L}}(y_i, f_{m-1}(x_i) + \gamma _m D(x_i;\theta )), \end{aligned}$$

where $y_i$ is the target variable, $f_{m-1}(x_i)$ is the prediction of the previous tree, and *N* is the number of training examples. The optimization is typically done using gradient descent, where the gradient of the loss function is computed with respect to the parameters of the tree.

Conventional GBDT is time-consuming when dealing with large amounts of data. LightGBM is an efficient and scalable implementation of GBDT that accelerates the training process while achieving decent accuracy. The computational cost of traditional GBDT is due to the way a decision tree is created. It implies scanning all the data instances to select a feature as a split point that maximizes the information gain. LightGBM [36] is introduced to overcome this limitation.

LightGBM uses a histogram-based technique to find the best-split points of decision trees by dividing continuous feature values into discrete bins and uses these bins to generate feature histograms during training. This method is more efficient in terms of memory use and training time than the GBDT algorithm. In contrast with numerous tree-based learning algorithms, such as XGBoost [37] that employed the level-wise tree growth, the LightGBM model splits the tree leaf-wise. The level-based tree growth approach involves a level-by-level expansion of the tree structure. The leaf-based tree growth technique allows the growth of an imbalanced tree based on the node that has the greatest reduction in loss. As a result, the training process may significantly speed up when the dataset is huge, since the leaf-wise tree method’s tree nodes are often smaller than the level-wise tree method’s tree nodes with the same tree depth.

LightGBM [36] developed two novel techniques which are gradient-based one-side sampling (GOSS) and exclusive feature bundling (EFB). GOSS exploits that data instances with large gradients are more significant in the computation of information gain. First, the instances are sorted by gradient, and then, GOSS picked a% instances with a large gradient and randomly selects b% from the remaining instances with a small gradient. Using GOSS, the amount of data instances is minimized while maintaining the accuracy of the constructed decision trees. Moreover, LightGBM reduces the number of features using EFB. Typically, high-dimensional features are quite sparse. Many features are mutually exclusive, which means that they can never have nonzero values at the same time. EFB can securely bundle these features into a single feature. This can considerably accelerate GBDT training without compromising performance. The proposed method steps are stated in Algorithm 1.
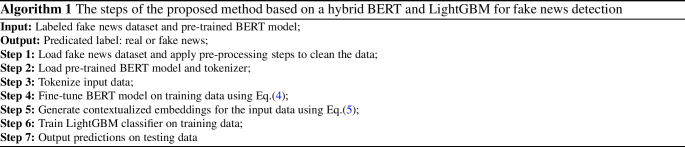


## Experimental results and discussion

### FakeNews datasets

ISOT dataset [13] contains 45,000 articles, almost evenly divided between fake and real news. The real articles are collected from the Reuters website, while fake articles are gathered from different untrusted websites indicated by Politifact. Each article’s title, full text, date, and subject are included in the dataset. Most of the articles are about politics and world news from 2016 to 2017.

The TI-CNN dataset [22] comprises 20,015 articles, including 8074 real news and 11,941 false news. The real articles are scraped from well-known reputable news websites, such as the New York Times and the Washington Post. The fake news was collected from over 240 websites on Kaggle. The dataset comprises a variety of information, including the title, text, author, and URL.

Fake News Corpus (FNC) [38] is a publicly available dataset made up of millions of news stories mostly collected from a curated list of 1001 domains. To better balance the dataset, extra New York Times and Webz.io (English-News-Articles) articles have been included, because the list does not include many trustworthy websites. The plain HTML data were processed to extract the article’s author, full text, title, and other additional fields. The corpus labels articles with a variety of tags, such as fake news, conspiracy theory, political, and credible. In this paper, we randomly sample 500,000 records from articles labeled as fake news and concatenate them with another 500,000 randomly sampled articles labeled as credible to create a balanced dataset of 1,000,000 articles to be used to train and test the proposed method.

### Evaluation metrics

We used five different evaluation metrics: accuracy (Acc $\%$), precision (Pre $\%$), recall (Rec $\%$), *F*1-score (*F*1 $\%$), and area under the receiver-operating characteristic curve (AUC).

Accuracy is the basic indicator for model evaluation, which describes the number of right predictions over all forecasts

8 $$\begin{aligned} {\text {Accuracy}}= \frac{{\text {TP}}+{\text {TN}}}{{\text {TP}}+{\text {TN}}+{\text {FP}}+{\text {FN}}}. \end{aligned}$$

Precision is a measure of what percentage of positive predictions were in fact correct

9 $$\begin{aligned} {\text {Precision}} = \frac{{\text {TP}}}{{\text {TP}}+{\text {FP}}}. \end{aligned}$$

Recall is measuring what percentage of true positives was successfully identified

10 $$\begin{aligned} {\text {Recall}} = \frac{{\text {TP}}}{{\text {TP}}+{\text {FN}}}. \end{aligned}$$

*F*1-Score is describing the harmonic mean of both precision and recall

11 $$\begin{aligned} F1 = \frac{2 \times {\text {Precision}}\times {\text {Recall}}}{{\text {Precision}}+{\text {Recall}}}. \end{aligned}$$

The receiver-operating characteristic (ROC) curve illustrates the trade-off between the true-positive rate and the false-positive rate at different threshold values. AUC summarized the performance of the classifier into a single measure by calculating the area under the ROC curve. The AUC value ranges from 0.5 to 1. A perfect predictor has an AUC score of 1, while a random guess predictor has an AUC score of 0.5.

**Fig. 2:**
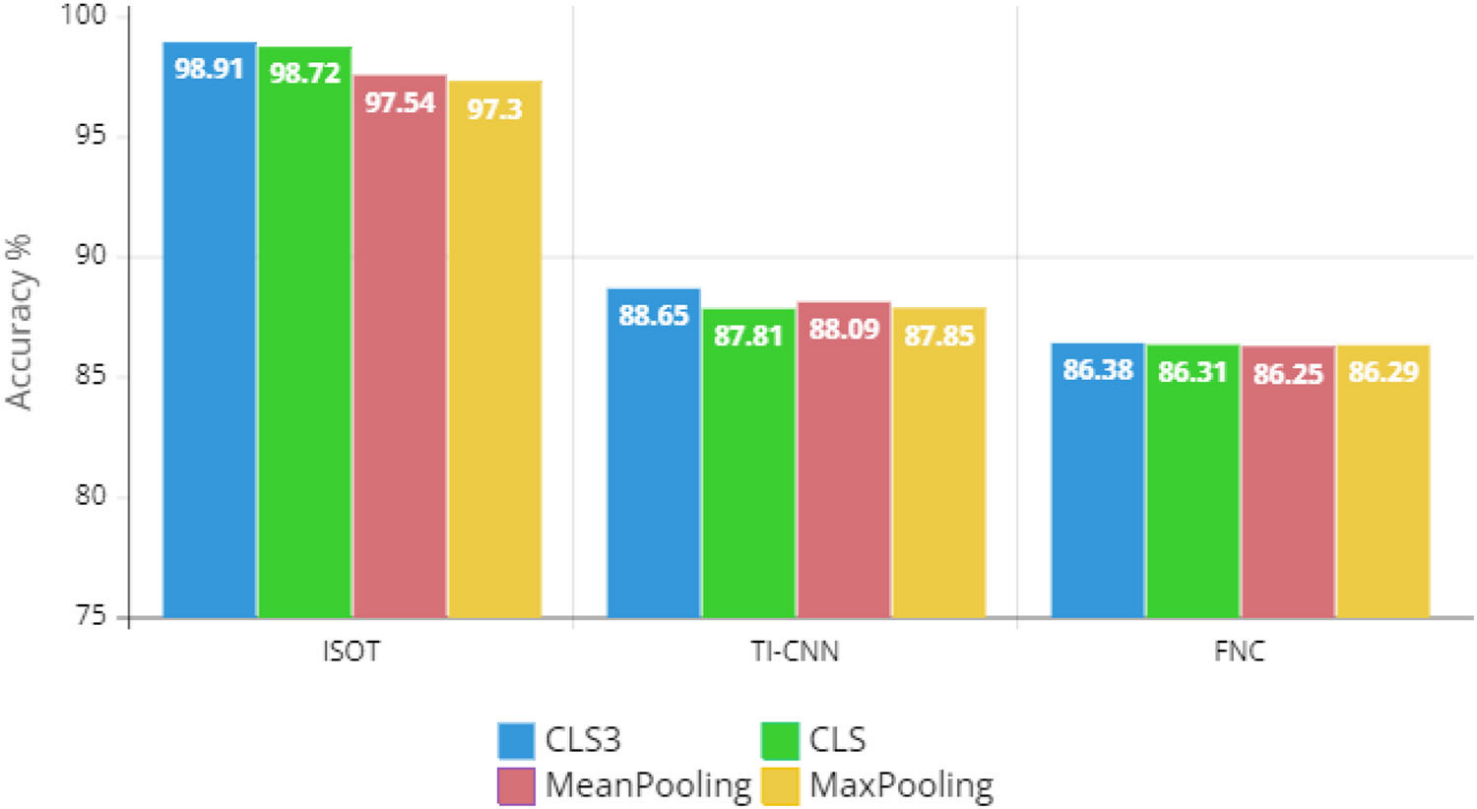
Comparison of hybrid LightGBM with different BERT-based embeddings for fake title classification on three different datasets

### Comparative analysis

We compare the proposed sentence embedding based on combining the $[{\text {CLS}}]$ embedding vectors of the last three layers (i.e., CLS3) to three different embedding methods, as shown in Fig. 2. The sentence embedding is extracted using one of the following three techniques: using only the last $[{\text {CLS}}]$ token embedding vector (i.e., CLS), taking the mean average of the sequence of hidden states at the output of the last layer of the model (i.e., MeanPooling), or taking the maximum across all hidden states of the last layer to get max pooling embeddings (i.e., MaxPooling). The proposed CLS3 shows better performance than the other embedding techniques. The average accuracy across the three datasets of the proposed CLS3 is 91.31 compared to 90.95, 90.63, and 90.48 for CLS, MeanPooling, and MaxPooling, respectively.

We compare the proposed method with different embedding techniques and different classification models. In literature, there are two popular embedding techniques: TF-IDF and GloVe. TF-IDF [39] is a statistical metric that assesses the relevance of a word to a document in a collection of documents or corpus. The value of TF-IDF increases proportionally to the number of occurrences of a word in the document and is mitigated by the number of documents containing the word, so that words that appear often in every text rank low, even though they appear frequently. GloVe [40] is an unsupervised learning technique that focuses on word co-occurrences statistics from a corpus. The co-occurrence matrix is used to infer semantic links between words; thus, the GloVe embeddings are related to the chances of two words appearing together. Different classification models have been used in comparison to the proposed method, including MNB, LR, LSVM, and LSTM, as discussed in the following sections.

### Implementation details

Each fake news dataset is randomly split into three independent sets: 60% for the training set, 30% for the testing set, and 10% for the validation set. The experiments were run on an Intel i9-10850K processor and a single GPU Nvidia TITAN Xp.

Machine-learning-based methods are implemented using the scikit-learn library [41]. Here, the LSTM model is constructed by one layer of bidirectional LSTM followed by a global max pooling layer and then the output layer. The number of hidden units is 100. Keras is used to implement LSTM-based model. We used the same LSTM architecture with both GloVe and BERT-based embeddings.

For BERT, we used “bert-base-uncased" pre-trained model from the Hugging face transformers library [42]. The BERT is fine-tuned on the fake news datasets for only one epoch using 1 cycle policy [43], a batch size of 6, and a learning rate of $5{\text {e}}^{-5}$. The hyperparameters of the LightGBM are automatically optimized using Optuna framework [44] on the validation set.

**Table 1:** Results on ISOT dataset for either title or full text

		Title				Text			
Embed	Model	Acc	*F*1	Pre	Rec	Acc	*F*1	Pre	Rec
TF-IDF	MNB	94.15	94.38	92.84	95.97	96.48	96.52	97.76	95.30
TF-IDF	LR	93.86	93.99	94.27	93.71	98.70	98.73	99.10	98.35
TF-IDF	LSVM	95.15	95.26	95.36	95.15	99.42	99.43	99.78	99.08
GloVe	MNB	89.79	89.16	97.79	81.93	83.74	83.65	86.22	81.23
GloVe	LSVM	94.32	94.30	97.06	91.68	91.82	92.08	91.32	92.85
GloVe	LSTM	97.94	97.98	98.40	97.57	98.45	98.48	98.85	98.12
BERT	LSTM	98.52	98.55	98.87	98.24	99.85	99.85	99.73	99.97
BERT	Proposed	98.91	98.94	99.03	98.84	99.88	99.88	99.79	99.97

**Table 2:** Results on TI-CNN dataset for either title or full text

Embed	Model	Title				Text			
		Acc	*F*1	Pre	Rec	Acc	*F*1	Pre	Rec
TF-IDF	MNB	85.66	88.48	84.28	93.12	90.10	91.99	88.20	96.13
TF-IDF	LR	86.26	88.94	84.87	93.41	92.72	93.85	93.79	93.90
TF-IDF	LSVM	87.20	89.09	89.83	88.35	94.51	95.26	97.29	93.32
GloVe	MNB	72.06	80.83	68.01	99.60	78.48	81.08	84.44	77.98
GloVe	LSVM	80.36	83.98	81.12	87.05	81.22	84.73	81.61	88.09
GloVe	LSTM	86.29	88.88	85.40	92.66	91.81	93.16	92.02	94.33
BERT	LSTM	87.52	89.71	87.54	91.99	96.31	96.89	96.53	97.25
BERT	Proposed	88.65	90.54	89.29	91.82	96.94	97.42	97.32	97.51

#### Results on the ISOT dataset

We compare the performance of the proposed method with other machine learning and deep-learning-based models that are using different word embedding techniques. Each method is tested to classify both the article’s title and the article’s full text to detect fake news, as shown in Table 1.

For TF-IDF embedding, the LSVM gives the highest performance (Accuracy = $95.15\%$, *F*1-score = 95.26%, and Precision = 95.36%, for title and Accuracy = 99.42%, *F*1-score = 99.43%, Precision = 99.78%, and Recall = 99.08% for text) compared to MNB and LR classifiers. MNB has obtained the lowest performance. Only recall for title classification of MNB (95.97%) is higher than the LSVM (95.15%) and LR (93.71%).

For GloVe embedding, The LSTM-based model achieves the highest performance (Accuracy = $97.94\%$, *F*1-score = 97.98%, Precision = 98.40%, and Recall = 97.57% for title and Accuracy = 98.45%, *F*1-score = 98.48%, Precision = 98.85%, and Recall = 98.12% for text) compared to MNB- and LSVM-based classifiers. However, the performance of TF-IDF + LSVM is higher than GloVe + LSTM for the full-text classification of fake news articles.

For BERT-based embedding, we compare the proposed method to the LSTM model based on the CLS embedding vector of the last hidden layer of BERT (BERT + LSTM). The performance of the proposed method (Accuracy = 98.91%, *F*1-score = 98.94%, Precision = 99.03%, and Recall = 98.84%) is better than the BERT + LSTM for title classification. For text classification, both methods give comparable results with a slight favor to the proposed method with an accuracy of 99.88% compared to 99.85% for the BERT + LSTM model.

Overall, the proposed method is ranked the highest performance for title and text results compared with the other machine-learning and deep-learning models.

#### Results on the TI-CNN dataset

Table 2 reports the result of the proposed method along with other machine-learning-based methods on the TI-CNN dataset using different word embedding techniques.

For TF-IDF embedding, LSVM gives the highest performance (for title classification, Accuracy = $87.20\%$, *F*1-score = $89.09\%$, and Precision = $89.83\%$, and for full-text classification, Accuracy = $94.51\%$, *F*1-score = $95.26\%$, and Precision = $97.29\%$) compared to MNB and LR classifiers. MNB has obtained the lowest performance.

For GloVe embedding, The LSTM-based model achieves the highest performance (Accuracy = $86.29\%$, *F*1-score = $88.88\%$, Precision = $85.40\%$ for title classification and Accuracy = $91.81\%$, *F*1-score = $93.16\%$, Precision = $92.02\%$, and Recall =$94.33\%$ for text classification ) compared to MNB- and LSVM-based classifiers. However, the performance of TF-IDF + LSVM is higher than GloVe + LSTM for both title and full-text classification of fake news articles.

**Table 3:** Results on FNC dataset for either title or full text

Embed	Model	Title				Text			
		Acc	*F*1	Pre	Rec	Acc	*F*1	Pre	Rec
TF-IDF	MNB	82.84	83.21	81.21	85.31	93.39	93.08	97.37	89.15
TF-IDF	LR	82.03	82.17	81.29	83.08	97.01	96.99	97.05	96.94
TF-IDF	LSVM	83.56	83.70	82.71	84.72	97.84	97.83	97.92	97.74
GloVe	MNB	59.88	54.99	62.38	49.16	71.96	71.43	72.58	70.32
GloVe	LSVM	68.07	67.10	68.98	65.32	85.81	85.45	87.36	83.62
GloVe	LSTM	81.63	81.65	81.30	82.00	96.12	96.11	96.07	96.16
BERT	LSTM	86.27	86.29	85.92	86.66	81.69	81.88	80.80	83.00
BERT	Proposed	86.38	86.33	86.36	86.31	99.06	99.05	99.07	99.04

For BERT-based embedding, the *F*1-score of BERT + LSTM for title and text classification are ($89.71\%$ and $96.89\%$) higher than TF-IDF + LSVM ($89.09\%$ and $95.26\%$). The performance of the proposed method (for title classification, Accuracy = $88.65\%$, *F*1-score = $90.54\%$, Precision = $89.29\%$, and Recall = $91.82\%$, for text classification, Accuracy = $96.94\%$, *F*1-score = $97.42\%$, Precision = $97.32\%$, and Recall = $97.51\%$) is better than the BERT + LSTM for both title and text classification.

Overall, the proposed method achieves superior performance for title and text results compared with the other machine-learning and deep learning models.

#### Results on the FNC dataset

The performance of the proposed method and all other machine learning-based methods on the FNC dataset is shown in Table 3.

For title classification, the proposed method gives the highest performance compared to all other methods. The accuracy, *F*1-score, and precision of the proposed method are $86.38\%$, $86.33\%$, and $86.36\%$ compared to $83.56\%$, $83.70\%$, and $82.71\%$ for TF-IDF + LSVM, $81.63\%$, $81.65\%$, and $81.30\%$ for GloVe + LSTM and $86.27\%$, $86.29\%$, and $85.92\%$ for BERT + LSTM. For the recall, the BERT + LSTM gives $86.66\%$ than the proposed method $86.31\%$.

For text classification, the proposed method achieves superior performance compared to all other methods. The accuracy, *F*1-score, precision, and recall of the proposed method are $99.06\%$, $99.05\%$, $99.07\%$, and $99.04\%$. compared to $97.84\%$, $97.83\%$, $97.92\%$, and $97.74\%$ for TF-IDF + LSVM, $96.12\%$, $96.11\%$, $96.07\%$, and $96.16\%$ for GloVe + LSTM and $81.69\%$, $81.88\%$, $80.80\%$, and $83.00\%$ for BERT + LSTM. The TF-IDF + LSVM is better than BERT + LSTM and gives the second-best performance after the proposed method.

**Fig. 3:**
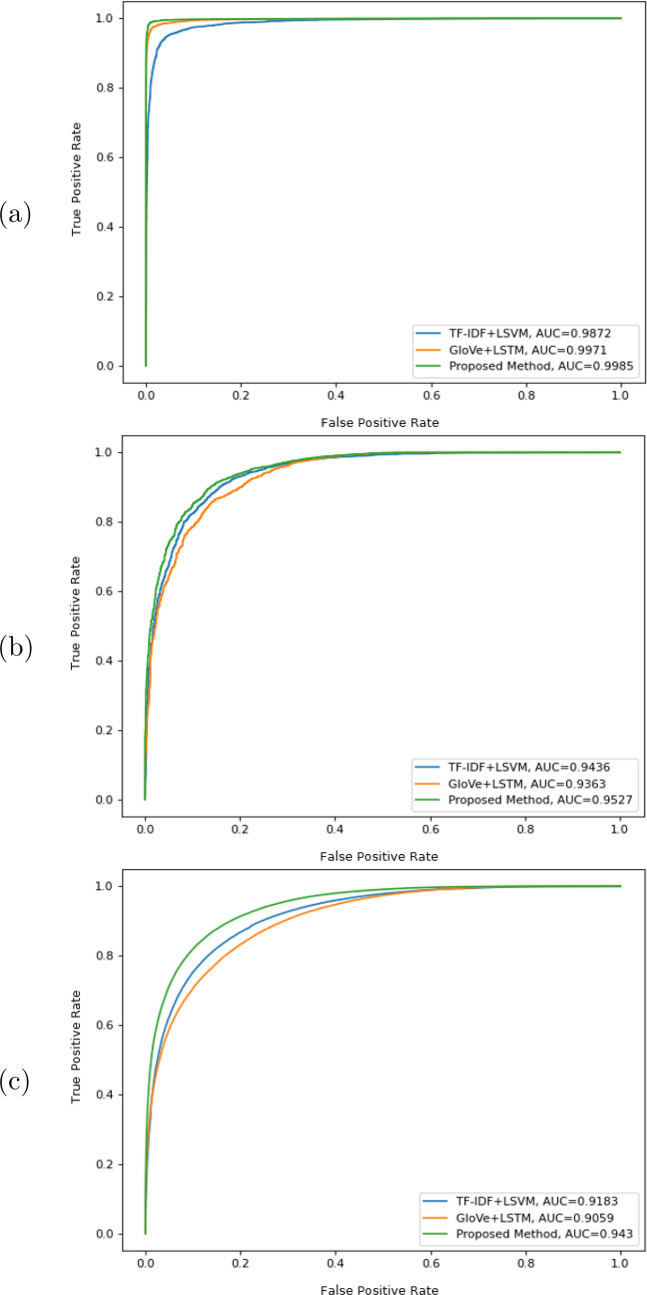
Comparison of ROC curves and AUC values of the proposed method, TF-IDF + LSVM, and GloVe + LSTM for fake title classification on different datasets. **a** ISOT dataset. **b** TI-CNN dataset. **c** FNC dataset

The ROC curves and AUC values of the proposed method, TF-IDF + LSVM, and GloVe + LSTM on ISOT, TI-CNN, and FNC datasets are illustrated in Fig. 3 for fake title classification. When the ROC curve is closer to the top-left corner, it indicates a better performance of the classifier. AUC measures the area under the ROC curve. As shown in Fig. 3, the proposed method is the superior model, since the area enclosed underneath the curve is the largest. The AUC of the proposed method on the ISOT, TI-CNN, and FNC datasets are 0.9985, 0.9527, and 0.9430, respectively, compared to 0.9872, 0.9436, and 0.9183 for TF-IDF + LSVM, and 0.9971, 0.9363, and 0.9059 for GloVe + LSTM.

**Table 4:** Comparison of the proposed methods with the state of the arts on different fake news datasets

Authors	Dataset	Embedding	Models	Acc	*F*1
Ahmed et al. [13]	ISOT	TF-IDF	SVM	92.00	–
Kansal [17]	ISOT	POS	XGBoost	92.32	91.85
Ozbay and Alatas [15]	ISOT	TF	DT	96.80	96.80
Nasir et al. [18]	ISOT	GloVe	CNNLSTM	99.00	99.00
Ozbay and Alatas [16]	ISOT	TF	GWO	99.50	99.70
Proposed method	ISOT	BERT	LightGBM	99.88	99.88
Yang et al. [22]	TI-CNN	Word2Vec	LSTM	–	87.58
Raj and Meel [23]	TI-CNN	GloVe	CNN	96.26	95.89
Proposed method	TI-CNN	BERT	LightGBM	96.94	97.42
Truică and Apostol [26]	FNC	BARTRoBERTa	LSTMCNN	92.50	–
Wijeratne [14]	FNC	TF-IDF	XGBoost	96.00	–
Wijeratne [14]	FNC	TF-IDF	SVM	97.00	–
Proposed method	FNC	BERT	LightGBM	99.06	99.05

#### Comparison with the-state-of-the-art methods

Table 4 shows a comparison between the proposed method and the state-of-the-art methods evaluated on ISOT, TI-CNN, and FNC datasets. The proposed method achieves superior performance compared to all the-state-of-art fake news classification methods. The proposed method has the highest accuracy on ISOT dataset with $99.88\%$ compared to $99.50\%$, $99.0\%$, $96.80\%$, $92.32\%$, and $92.00\%$ for TF + GWO, GloVE + CNNLSTM, TF-IDF + DT, POS + XGBoost, and TF-IDF + SVM methods, respectively.

For the TI-CNN dataset, the proposed method has the best *F*1-score with $97.42\%$ compared to $97.42\%$, and $87.58\%$ for GloVE + CNN and Word2Vec + LSTM methods, respectively. For the FNC dataset, the accuracy of the proposed method is surpassing other methods. It achieves $99.06\%$ compared to $97.00\%$, $96.00\%$, and $92.50\%$ for TF-IDF + SVM, TF-IDF + XGBoost, and BARTRoBERTa + LSTMCNN methods, respectively.

Our proposed method has worked better than other methods for fake news detection for many reasons, including that BERT is pre-trained on large amounts of text data, making it capable of capturing complex patterns and relationships in language. The combination of BERT and LightGBM allows us to leverage the strengths of both methods. BERT can capture complex semantic information from text, while LightGBM can handle high-dimensional feature spaces and optimize complex objective functions.

While the BERT model itself is computationally intensive due to its large number of parameters (110*M*), we have only used it as a feature extractor to obtain contextualized embeddings for each input text. This significantly reduces the computational complexity of the overall model as the BERT model only needs to be fine-tuned on the fake news dataset. The embeddings are then fed as input to LightGBM, which is trained in a parallel and memory-efficient manner, which further reduces the computational complexity of the overall model.

## Conclusion

In this paper, a novel hybrid model is proposed for automatically detecting false news. The proposed method combines BERT and LightGBM models to form an effective classification system. The LightGBM model is built on top of a pre-trained BERT word embedding model. The results show that the proposed method produces more accurate results, with an accuracy of 99.88%, 96.94%, and 99.06% on ISOT, TI-CNN, and FNC datasets, respectively. The proposed hybrid method outperforms different combinations of word embedding techniques and classification approaches, as well as current state-of-the-art methods on different real-world fake news datasets.

## Data Availability

The data used in the paper are available on request from the corresponding author.
